# Antimalarial Property and Acute Toxicity of the Leaves of *Theobroma cacao* L.

**DOI:** 10.1155/2021/2852442

**Published:** 2021-07-13

**Authors:** Gustav Komlaga, Arnold Donkor Forkuo, Nadiatu Suleman, Desmond Nkrumah, Reinhard Nketia, Samuel Oppong Bekoe

**Affiliations:** ^1^Department of Pharmacognosy, KNUST, Kumasi, Ghana; ^2^Department of Pharmacology, KNUST, Kumasi, Ghana; ^3^Department of Pharmaceutical Chemistry, KNUST, Kumasi, Ghana

## Abstract

The leaf of *Theobroma* cacao L. is used in traditional medicine in Ghana for the treatment of malaria, yet, with no scientific evidence of its antimalarial property in animals. It was, therefore, studied to validate the antimalarial property in *Plasmodium berghei-*infected mice. Infected mice were treated with an aqueous extract of *T. cacao* leaf at different doses of 100, 200, and 400 mg/kg daily for four days. Parasitaemia was determined before treatment and 24 hours following the last dose of extract. The % reduction in parasitaemia and ED_50_ and ED_90_ of the extract were determined. Body weight, rectal temperature, and daily mortality of mice were also recorded. The extract had ED_50_ and ED_90_ of 242.20 ± 29.38 and 351.00 ± 29.52 mg/kg/day, respectively. Percentage parasitaemia suppression was significant for all doses. The extract at the maximum dose of 400 mg/kg body weight had the highest % parasitaemia suppression of 79.19%; mean survival time of 24.00 ± 2.19 days and median survival of 23 days; body weight increase of 3.82 ± 0.59; and the lowest body temperature reduction of 0.79 ± 0.11°C. *T. cacao* leaf extract showed an antimalarial property in *P. berghei-*infected mice. This reinforces the justification for the use of the plant material in treating malaria in Ghana.

## 1. Introduction

Malaria, an infectious disease borne by mosquitoes, is a major public health burden globally. This is irrespective of the successes achieved worldwide in reducing the estimated prevalence of the disease by 9 million between 2000 and 2019 [1]. Globally, an estimated 1.5 billion malaria cases and 7.6 million malaria deaths have been averted in the period of 2000–2019. Most of the cases (82%) and deaths (94%) averted were in the World Health Organization (WHO) African Region [1]. However, WHO African Region, with an estimated 215 million cases in 2019, accounted for about 94% of cases [1]. Due to many factors, including cultural practices, cost, and sometimes unavailability of first-line treatments, many Ghanaians, and by extension, Africans, use medicinal plants for the treatment of the disease. One such medicinal plant is *Theobroma cacao*.

*T. cacao*, commonly called cocoa, is a small evergreen tree native to South America but domesticated in Ghana and other African countries. It is an important crop in that it provides livelihood in terms of food, income, employment, industrial raw materials, and resources for poverty reduction for smallholder farmers in Ghana. Furthermore, it provides raw materials for the multibillion global chocolate industry [2]. The seed is used to make cocoa powder and chocolate. The plant is economically important as cocoa butter from the seeds is widely used in the manufacture of chocolates, beverages, ice creams, and desserts [3, 4]. The seeds also contain polyphenols and flavonoids that possess myriad health benefits [4].

The leaf has many uses in traditional medicine. The decoction is a remedy in the traditional treatment of malaria [5, 6]. The leaf is also used to treat asthma, weakness, diarrhea, fractures, loss of appetite, parasites, pneumonia and cough, colic, and poisoning. In a previous study, the *in vitr*o antiplasmodial activity of the aqueous extract of the leaf was established [6]. It was, however, not clear to what extent the *in vitro* activity could translate into malaria treatment in animals. In this study, the antimalarial property of the aqueous extract of the leaf was evaluated in *Plasmodium berghei-*infected mice.

## 2. Materials and Methods

### 2.1. Equipment and Reagents

A light microscope (Leica DM750 HD microscope, Wetzlar, Germany), slide and coverslip, and weighing scale were used.

### 2.2. Plant Material Collection

Fresh leaves of *Theobroma cacao* were harvested in October 2019 at The Physique Garden of the Faculty of Pharmacy and Pharmaceutical Sciences at the Kwame Nkrumah University of Science and Technology, Kumasi. It was authenticated by Prof. Gustav Komlaga of the Department of Pharmacognosy. Herbarium specimen (KNUST/HM1/2014/L004) was deposited at the Herbarium of the Department of Herbal Medicine, Faculty of Pharmacy and Pharmaceutical Sciences, Kwame Nkrumah University of Science and Technology (KNUST), Kumasi.

### 2.3. Plant Material Processing

Debris and foreign matter were manually removed from the leaves. They were then washed under running water, cut into smaller sizes, and shade-dried for two weeks. The dried material was milled into coarse powder with a mechanical grinder and kept until needed for use.

### 2.4. Plant Material Extraction

The dried powdered leaf (0.2 kg) was boiled in 1.5 L of water on an electric stove for 30 minutes. It was allowed to cool and filtered with cotton wool followed by Whatman filter paper No. 1 (Whatman®, England). The extract was freeze-dried at the Council for Scientific and Industrial Research (CSIR), Fumesua, Kumasi. The dried extract was labelled and kept in the desiccator until required for use.

### 2.5. Phytochemical Screening

The leaf powder was screened for phytochemical constituents following standard procedures [7].

### 2.6. Experimental Animals

Six (6) Swiss albino rats (140–145 g) were used for the acute toxicity studies. Twenty-five (25) healthy adult Swiss albino mice of both sexes (20–28 g and 6–8 weeks of age) were used for antiplasmodial studies. All animals were obtained from the Noguchi Memorial Institute for Medical Research (NMIMR), University of Ghana, Legon, Accra, Ghana. Animals were housed under standard conditions at the animal house of the Department of Pharmacology, KNUST, in plastic cages with softwood shavings and chips as beddings. They were exposed to a 12/12 house dark-to-light cycle and provided with free access to pellet diet and clean drinking water. Animals were allowed to adapt to the room environment for at least one week before the beginning of the experiments [8]. The institutional standard operating procedure for animal research was strictly observed [9] and ethical approval with ID number KNUST 001 was granted by the KNUST Animal Research Ethics Committee.

### 2.7. Parasites and Hosts

Blood-stage samples of the chloroquine-sensitive ANKA strains of rodent *Plasmodium berghei,* kept in liquid nitrogen at the Central lab of KNUST, were used in the antiplasmodial experiment. The parasites were passaged in three Swiss albino mice used as parasite donors. The donor mice were euthanized with diethyl ether, and the blood was collected by cardiac puncture into a heparinized vacutainer tubes. The infected blood (parasitaemia 30%) was diluted with physiological saline (0.9%) such that 1 ml blood contained 1 × 10^7^ parasitized erythrocytes [10]. An aliquot of 0.2 ml corresponding to 2 × 10^6^ infected red blood cells (RBCs) of this suspension was intraperitoneally (i. p.) administered to the mice for the antiplasmodial assay. All surviving infected experimental mice were euthanized at the end of the experiment on day 30.

### 2.8. Acute Toxicity Study

The acute toxicity of the extract was assessed according to OECD guidelines 425 [11] in seven-to-eigh week-old healthy nulliparous and nonpregnant female Swiss albino rats (six in number). Because *T. cacao* leaf has a long history of use in traditional medicine as an antimalarial material without demonstrable toxicity, the limit test at the single dose level of 2000 mg/kg body weight was adopted. An aqueous solution of the crude extract was administered by gavage using a stomach tube. Animals were fasted overnight but were allowed unlimited access to water prior to dosing. After the period of fasting, the rats were weighed and administered given volumes of the test solution based on their weights. After extract administration, food was withheld for a further three hours, during which the rats were individually observed at 30 min intervals for the first 1 h and then occasionally for the next 24 h with special attention given in the first 4 h. Thereafter, observation was made daily for the next 13 days for manifestation of signs of toxicity, including gross physical and behavioral changes such as rigidity, sleepiness, diarrhoea, abnormal secretion or hair erection, and death. Rats were also weighed on day seven and day 14 of the experiment.

### 2.9. Curative Activity (Rane's Test)

The antimalarial therapeutic activity of the crude extract was assessed using Peters' four-day curative test in mice [12]. Infected mice were randomly placed into five groups of five. Animals were administered, by oral gavage, the extract or vehicle, 72 h (day 3) after the infection and thence every 24 h for three consecutive days. Artesunate (Ar) was used as the reference drug. Two groups, assigned negative and positive controls, received 2 ml normal saline and artesunate (4 mg/kg/day) orally (p. o.), respectively. The remaining three groups were dosed with 100, 200, and 400 mg/kg of the extract dissolved in normal saline. Parasitaemia was determined using the light microscope on the day of first treatment (three days after the infection) and 24 hours following the last dose (seven days after the infection). Giemsa-stained thin blood smears were prepared from the tail of each mouse, and the number of both parasitized and nonparasitized erythrocytes was counted in five randomly selected fields under a magnification of ×100 objective lens of a light microscope (Leica DM750 HD Microscope, Wetzlar, Germany). The drug concentrations, which reduced the parasitaemia by 50% (ED_50_) and 90% (ED_90_), were, respectively, determined. Moreover, the percentages by which each dose suppressed parasite growth were determined on day seven. Body weight and rectal temperature of mice were taken on days three and seven following infection [13]. The animals were monitored daily for mortality, starting from the day of infection to the 30^th^ day after the infection, to determine the mean survival time (MST). MST is the period in which infected mice survived in the experiment. Deaths, if any, were recorded during the period, and the MST was calculated for each group.

The percentage parasitaemia was calculated as follows:

(1) $$\begin{array}{c} \%\text{parasitaemia}=\frac{\text{number of parasitized RBC}}{\text{total number of RBC counted}}\times 100. \end{array}$$

Percentage chemosuppression was calculated as follows:

(2) $$\begin{array}{c} \frac{A-B}{A}\;x100, \end{array}$$

where *A* is the percentage parasitaemia in the negative control group and *B* is the percentage parasitaemia in the test group.

The mean survival time (MST) for each group was calculated as follows:

(3) $$\begin{array}{c} \text{MST}=\frac{\left(\sum \text{survival times of mice}/\text{group}\right)}{\text{number of mice in the group}}. \end{array}$$

### 2.10. Data Analysis

Data were analyzed using Graph Pad Prism (GraphPad Software version 8.0.2, San Diego, CA, USA) and presented as mean ± SD. Comparisons were made between the negative control group and treatment groups as well as the positive control group using one-way analysis of variance (ANOVA) followed by Dunnet's multiple comparison tests. Rectal temperature and body weight before and after infection and treatment were compared using two-way ANOVA followed by Dunnet's multiple comparison tests. Survival curves were compared under log-rank (Mantel–Cox) test, and the differences were compared at *p* value < 0.0001.

## 3. Results

### 3.1. Phytochemical Analysis

The powdered leaves tested positive to all phytoconstituents tested but sterols.

### 3.2. Acute Oral Toxicity Assessment

The acute toxicity study of the extract in rats revealed no gross physical and behavioral changes such as rigidity, sleepiness, diarrhoea, abnormal secretion, or hair erection for 24 h. All rats survived the two-week observation period at the dose level of 2000 mg/kg body weight. No significant changes in body weight of rats were observed except that due to normal growth (Table 1).

### 3.3. Curative Study of the Extract (Rane's Test)

There was a significant (*p* < 0.0001) suppression of parasitaemia at all dose levels in drug-treated groups on day seven following infection. The highest suppression of parasitaemia was 79.19% in mice treated with 400 mg/kg/day of extract (Table 2). ED_50_ and ED_90_ of the extract were 242.20 ± 29.38 and 351.00 ± 29.52 mg/kg/day, respectively.

**Figure 1:**
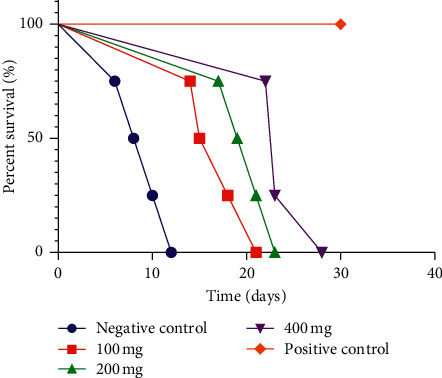
Survival curve of experimental mice in the curative test. The survival curves are significantly different at a *p* value < 0.0001 according to log-rank (Mantel–Cox) test.

Survival time was extended among the treated groups in a dose-dependent trend. The survival curve of the infected mice is shown in Figure 1. The tick mark for censored mice (positive control) is indicated by an orange dot on the PC graph. All four members of PC (positive control) group survived until the end of the study (30 days). In contrast, no subjects remained in the 100 mg group after days 21, the 200 mg group had no mice left after 23 days, and the 400 mg group had no remaining mice after day 28 following infection. For the negative control group, there were no remaining mice after 12 days. The mean survival time for 100 mg group was 17.00 ± 2.92, and the median survival was 16.5. The 200 mg group had a mean survival time of 20.00 ± 2.93 days and median survival of 20 days, and the 400 mg group had a MST of 24.00 ± 2.19 and median survival of 23 (Table 3). Alternately, the negative control had a MST of 9.00 ± 0.63 and median survival of nine days, while the MST of the positive control group is unknown but beyond 30 days and its median survival is undefined.

**Figure 2:**
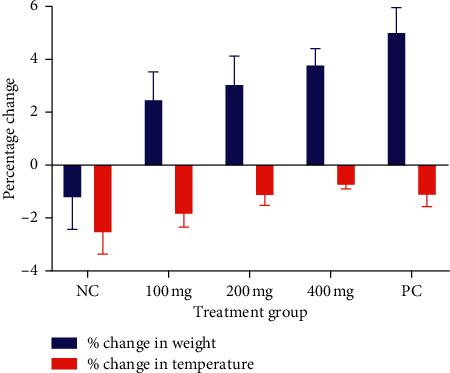
A graph showing loss or gain in body weight and body temperature.

Treated groups significantly (*p* < 0.05) increased in body weight (Figure 2). Moreover, the falling body temperature observed in infected mice was upended upon treatment with the extract (Figure 2).

## 4. Discussion

The aqueous extract of *T. cacao* leaves, in a previous study, demonstrated *in vitro* antiplasmodial activity against both chloroquine-sensitive 3D7 and chloroquine-resistant W2 strains of *P. falciparum*. It also did not present any toxicity against human umbilicus vein endothelial cells (HUVECs) [6]. Based on the aforementioned results, with regard to its traditional use for treating malaria, the current project was conceived.

Plasmodium infection, when uncontrolled, leads to increased parasitaemia and subsequent development of various symptoms, including malaise, fever characterized by elevated body temperature, loss in body weight due to loss of appetite, anaemia, and eventually death [14]. Treatment, however, leads to amelioration of the symptoms, thus preventing their deterioration and, ultimately, death.

Parasitaemia was the main factor considered in this study. However, body temperature changes, body weight loss, and death were also monitored.

The antimalarial property of the aqueous extract of *T. cocoa* leaves following *P*. *berghei* infection in mice was demonstrated by the significant (*p* < 0.0001) parasitaemia suppression, increased mean survival time (MST) and median survival, reversal of weight loss, and upturn of the falling body temperature compared with the untreated group (Tables 1 and 4). The extract had ED_50_ and ED_90_ of 242.20 ± 29.38 and 351.00 ± 29.52 mg/kg/day, respectively. The suppression of parasitaemia agrees with the *in vitro* antiplasmodial activity demonstrated by the *T. cacao* leaf extract [6], where the aqueous extract displayed moderate but similar activity against both chloroquine-sensitive 3D7 and chloroquine-resistant W2 strains of *P. falciparum*. Together, these observations suggest the potential usefulness of the plant material in the treatment of malaria, though the study did not result in complete parasite eradication.

The percentage suppression in parasitaemia shows the level of reduction in parasite load as a result of treatment, and it indicates the extent to which substances may have relieved the host of the effect or symptoms of the infection. In this case, weight loss was reversed and the treated animals regained significant (*p* < 0.05) weight. This observation could only be attributed to the restoration of the animals' appetite as a result of extract treatment. According to Dascombe and Sidara [15], loss of appetite is a common phenomenon in *Plasmodium berghei* infection of mice, and this has been attributed to increased turnover in the brain of the neurotransmitter, 5-hydroxytryptamine (5-HT, serotonin). The restoration of food intake observed in the mice could, therefore, be the result of the extract's effect on the brain in ways that might have reversed the changes in the levels of 5-HT.

*Plasmodium berghei* infection in mice, instead of causing fever and, therefore, hyperthermia as in humans, results in hypothermia. According to literature [15], such observation is typical in laboratory models of human malaria. The fall in temperature was explained by the general debilitating effect of the infection, such as anaemia, on heat production and/or heat conservation in small animals and reduced metabolism in infected mice [15].

The progressing hypothermia associated with *P. berghei* infection in mice was also upended in treated animals in a dose-dependent manner. The halt in falling body temperature could be explained by increased animal activity as they (treated animals) recover from the infection. These and similar changes in the physiology of the treated mice subsequently lead to improvement in their health conditions and hence their extended mean survival time and median survival.

The acute toxicity study showed no observable adverse effects in the tested animals with the extract having LD_50_ above 2000 mg/kg body weight. This suggests its safety to animals. Indeed, the aqueous extract at a dose range of 0.78–100 *μ*g/mL demonstrated no cytotoxicity when studied in human umbilical vein endothelial cells (HUVECs) and has a high selectivity index [6]. These observations, thus, offer the potential for safer therapy.

The phytochemical screening of *T. cacao* leaves revealed the presence of tannins, flavonoids, reducing sugars, saponins, alkaloids, coumarins, and sterols. These findings agree with findings in such previous study [6]. Various compounds belonging to different classes of phytochemicals as observed in this study have demonstrated antiplasmodial activity [16]. Individual members of these classes of compounds revealed here could, therefore, be responsible for the antimalarial activity observed in this study.

## 5. Conclusion

In conclusion, the aqueous extract of *T. cacao* leaves has shown a remarkable antiplasmodial activity and potential antimalarial property in *P. berghei*-infected mice. This observation largely reinforces the justification for the use of cocoa leaves in the traditional treatment of malaria in Ghana.

## Figures and Tables

**Table 1:** Body weight changes of rats over 14 days following the administration of a single 2000 mg/kg body weight dose of extract.

Mean body weight on day 0 (g)	Mean body weight on day 7 (g)	Mean body weight on day 14 (g)	Mean change in body weight on day 7 (g)	Mean change in body weight on day 14 (g)

139.9 ± 1.44	151.1 ± 1.39	160.9 ± 1.37	11.18 ± 0.34	21.00 ± 1.23

**Table 2:** Parasitaemia levels and percentage parasitaemia suppression in *P. berghei*-infected mice.

Dose (mg/kg/day)	Parasitaemia (%) on day 3 after the infection	Parasitaemia (%) on day 7 after the infection	% suppression
100	66.12 ± 6.36	34.51 ± 5.50^a^	59.98 ± 6.05^a^
200	65.41 ± 4.17	21.01 ± 1.95^a^	67.52 ± 1.68^a^
400	64.43 ± 3.89	17.97 ± 3.36^a^	79.16 ± 3.90^a^
NC	69.68 ± 4.20	86.23 ± 2.18	NS
PC	66.43 ± 6.98	11.75 ± 1.19^a^	86.37 ± 0.61^a^

Values were presented as mean ± SEM; *n* = 4; NC = vehicle-treated group; Ar = artesunate; NS = no suppression. Values are significantly different at ^p^ < 0.0001.

**Table 3:** Survivability indicators of infected mice.

Dose (mg/kg/day)	MST (days)	Median survival (days)
100	17.00 ± 2.92	16.5
200	20.00 ± 2.93c	20
400	24.00 ± 2.19^b^	23
NC	9.00 ± 0.63	9
PC	>30.00 ± 0.00^a^	Undefined

Values were presented as mean ± SEM; *n* = 4; NC = vehicle-treated group; PC = positive control group; MST = mean survival time. Values are significantly different at ^a^*p* < 0.0001, ^b^*p* < 0.001, and ^c^*p* < 0.01.

**Table 4:** Phytochemical analysis of powdered leaves.

Phytoconstituents	Result
Tannins	+
Flavonoids	+
Reducing sugars	+
Saponins	+
Alkaloids	+
Triterpenoids	+
Sterols	−
Coumarins	+

+: detected; −: not detected.

## Data Availability

All data in relation to this study are included within the manuscript.
